# β-Actin and γ-Actin Are Each Dispensable for Auditory Hair Cell Development But Required for Stereocilia Maintenance

**DOI:** 10.1371/journal.pgen.1001158

**Published:** 2010-10-14

**Authors:** Benjamin J. Perrin, Kevin J. Sonnemann, James M. Ervasti

**Affiliations:** Department of Biochemistry, Molecular Biology, and Biophysics, University of Minnesota, Minneapolis, Minnesota, United States of America; University of Washington, United States of America

## Abstract

Hair cell stereocilia structure depends on actin filaments composed of cytoplasmic β-actin and γ-actin isoforms. Mutations in either gene can lead to progressive hearing loss in humans. Since β-actin and γ-actin isoforms are 99% identical at the protein level, it is unclear whether each isoform has distinct cellular roles. Here, we compared the functions of β-actin and γ-actin in stereocilia formation and maintenance by generating mice conditionally knocked out for *Actb* or *Actg1* in hair cells. We found that, although cytoplasmic actin is necessary, neither β-actin nor γ-actin is required for normal stereocilia development or auditory function in young animals. However, aging mice with β-actin– or γ-actin–deficient hair cells develop different patterns of progressive hearing loss and distinct pathogenic changes in stereocilia morphology, despite colocalization of the actin isoforms. These results demonstrate overlapping developmental roles but unique post-developmental functions for β-actin and γ-actin in maintaining hair cell stereocilia.

## Author Summary

Genetic mutations that cause progressive hearing loss offer insight into the cellular processes that are required to maintain auditory function. In humans, several such deafness-causing mutations have been identified in the gene encoding γ-actin. This protein, together with the closely-related β-actin protein, comprise the primary structural elements of stereocilia, which are specialized structures on sensory cells in the inner ear that convert mechanical sound energy into neural signals. β-actin and γ-actin are 99% identical, but their slight differences have been exactly conserved through evolution from birds to mammals suggesting that each protein may have important and distinct functions. We tested this idea by knocking out each gene in mouse auditory hair cells. Although the isoforms are completely redundant during stereocilia development, β-actin and γ-actin have different functions during stereocilia maintenance. Both knockout mice had normal hearing at young ages, but developed specific types of progressive hearing loss and stereocilia pathology that differed depending on which isoform was lost. These separate maintenance pathways are likely important for maintaining auditory function during aging and may contribute to future understanding of common forms of age-related hearing loss in humans.

## Introduction

Actin reversibly polymerizes to form stiff, strong and polarized filaments, a process that is modulated by numerous actin binding proteins. Polymerization itself can provide force for diverse functions including membrane protrusion and cell motility, endocytosis or propelling objects within the cytoplasm. Actin filaments are substrates for myosin motors and also form structural elements of certain cellular organelles including microvilli and stereocilia.

*Actb* and *Actg1* encode β-actin and γ-actin, respectively, which are the two ubiquitously expressed cytoplasmic members of the actin family that includes four additional genes that are predominately expressed in muscle [1]. β-Actin and γ-actin are closely related proteins, with each amino acid sequence exactly conserved across vertebrates. The isoforms differ by only 4 biochemically-similar residues clustered in the N-terminal 10 amino acids [2]. However, these subtle differences were recently shown to confer distinct biochemical properties, with β-actin exhibiting more dynamic polymerization properties than γ-actin [3]. Interestingly, β-actin and γ-actin can co-polymerize with the overall dynamics reflecting the composition of the mixture [3]. In addition to these biochemical differences, β-actin and γ-actin are reported to have different localization patterns in certain cell types [4], [5] and are subject to different post-translational modifications [6]. Finally, more severe phenotypes result from loss of β-actin than γ-actin *in vivo*. Mice lacking β-actin are embryonic lethal [7], [8] while *Actg1* knockout mice (*Actg1^−/−^*) are viable but develop progressive hearing loss [9]. In humans, a mutation in *ACTB* results in severe syndromic phenotypes that include developmental malformations and deafness [10]. In contrast, several different mutations in human *ACTG1* cause dominant progressive hearing loss without other syndromic phenotypes [11]–[15].

Auditory function seems to be particularly sensitive to perturbations of cytoplasmic actins, perhaps because actin is a key structural component of auditory hair cells, which convert sound waves to neural signals. Hair cells are housed in the organ of Corti, both of which feature an intricate architecture that is required for proper function. The organ of Corti consists of three rows of outer hair cells (OHCs) and one row of inner hair cells (IHCs) together with several types of support cells. This ribbon-like structure runs longitudinally along the length of the cochlea. OHCs function to improve sensitivity to sound while IHCs are the auditory receptors [16]. Both cell types are topped with specialized structures called stereocilia, which are elaborated microvilli formed from a mixture of β-actin and γ-actin filaments that are organized in a tightly bundled paracrystalline array [17]–[20]. In mouse OHCs, around 90 stereocilia are arranged in three staircase-like rows within a V-shaped bundle. Stereocilia are connected by a variety of proteinaceous links, including tip links that connect the tops of stereocilia in shorter rows to the sides of stereocilia in the adjacent taller row (reviewed in [21]). Tip links are composed of cadherin23 and protocadherin15 and are required for mechanoelectric transduction that occurs when sound pressure waves displace the hair bundle [22], [23]. Hair cells, together with their stereocilia, are not regenerated and must therefore be preserved for the life of the organism. Hearing maintenance is affected by the composition of stereocilia and tip links. Dimorphic alleles of the gene encoding cadherin23 confer either susceptibility or resistance to age-related hearing loss in several different inbred mouse strains [24], [25].

We previously demonstrated that whole body *Actg1* ^−/−^ mice initially have normal hearing and stereocilia morphology while young. However, these mice develop progressive hearing loss together with stereocilia degradation [9]. Therefore, γ-actin is dispensable for stereocilia formation but is required for maintenance. Here, we used conditional gene ablation in mice to show that hair cell stereocilia development requires at least one cytoplasmic actin, but proceeds normally in the absence of either isoform. Mice with β-actin or γ-actin deficient hair cells each have normal hearing at young ages but subsequently develop distinct forms of adult-onset, progressive hearing loss along with different pathogenic changes in stereocilia morphology. Fluorescently-labeled primary antibodies specific for β-actin or γ-actin each uniformly label the stereocilia actin core. Together, these data show that β-actin and γ-actin, despite exhibiting nearly identical protein sequences and inner hair cell stereocilia localization patterns, perform distinct functions in maintaining auditory hair cell stereocilia.

## Results

### β-actin is not required for stereocilia formation

**Figure 1:**
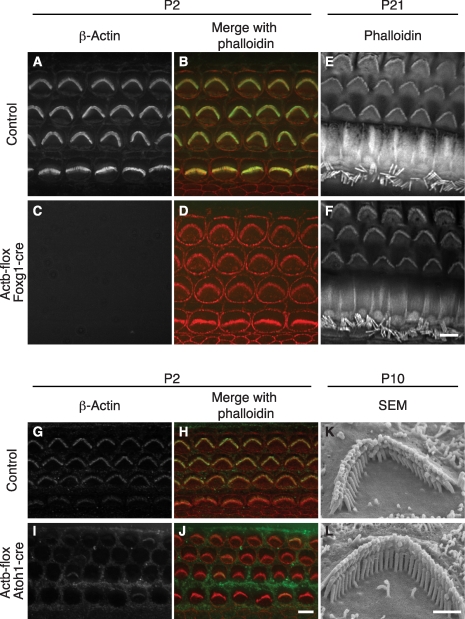
β-actin is not required for stereocilia formation. (A–F) Hair cells from control or *Actb*-flox *Foxg1*-cre mice. β-Actin staining of stereocilia in control pup overlays with phalloidin staining (A–B), but is absent from all cell types in *Actb*-flox *Foxg1*-cre mice (C) while phalloidin staining of stereocilia appears normal (D). At P21, phalloidin stained stereocilia are similar in control (E) and *Actb*-flox *Foxg1*-cre (F) hair cells. (G–L) Analysis of *Actb*-flox *Atoh1*-cre hair cells. In control hair cells (G–H), anti-β-actin stained stereocilia overlay with phalloidin staining. In *Actb*-flox *Foxg1*-cre hair cells β-actin is stained in support cells but is mostly absent from hair cell stereocilia (I), while phalloidin staining reveals normal stereocilia (J). (K–L) SEM analysis shows that control (K) and *Actb*-flox *Atoh1*-cre (L) stereocilia have similar architecture. In merged images, phalloidin staining is red and β-actin is green. Bar in A–J is 5 µm; K–L is 1 µm.

β-actin might be essential for stereocilia development because it is the only cytoplasmic actin detected at the earliest stages of stereocilia formation [9]. To test this idea, we used *Foxg1*-cre in combination with a newly generated floxed allele of *Actb* (Figure S1) to knock out β-actin in the developing ear. Consistent with the early onset of *Foxg1*-cre, which is expressed throughout the otic vesicle from E9.5 [26], *Actb*-flox *Foxg1*-cre inner ear tissue at postnatal day 2 (P2) was devoid of β-actin as detected by immunofluorescent staining (Figure 1A and 1C). Surprisingly, phalloidin staining revealed stereocilia with apparently normal morphology and organization (Figure 1B and 1D). Furthermore, phalloidin-stained stereocilia were still detectable at P21 (Figure 1E and 1F).

Because *Actb*-flox *Foxg1*-cre mice rarely survived to adulthood, we also used *Atoh1*-cre to mediate *Actb* knockout. In the organ of Corti, *Atoh1*-cre expression is limited to IHCs and OHCs with expression beginning before E18.5 [27]. Correspondingly, β-actin immunofluorescent staining is retained in neighboring support cells but is largely absent from both inner and outer hair cells (Figure 1G and 1I). Residual β-actin expression at early postnatal stages is likely due to a long β-actin protein half-life. Consistent with our observations in *Actb*-flox *Foxg1*-cre mice, *Actb*-flox *Atoh1*-cre stereocilia appear to develop normally as judged by phalloidin staining at P2 (Figure 1H and 1J), as well as by scanning electron microscopy (SEM) at P10 (Figure 1K and 1L).

### β-actin and γ-actin colocalize

β-actin and γ-actin have been shown to have distinct localization patterns in a variety of cell types [4], [5], [9], [18], [28]; however, owing to species differences or experimental design, conflicting localization patterns have been reported. Here, we assessed whether primary-secondary antibody combinations report the same localization pattern as dye-conjugated primary antibodies when staining the densely packed actin array of IHC stereocilia.

**Figure 2:**
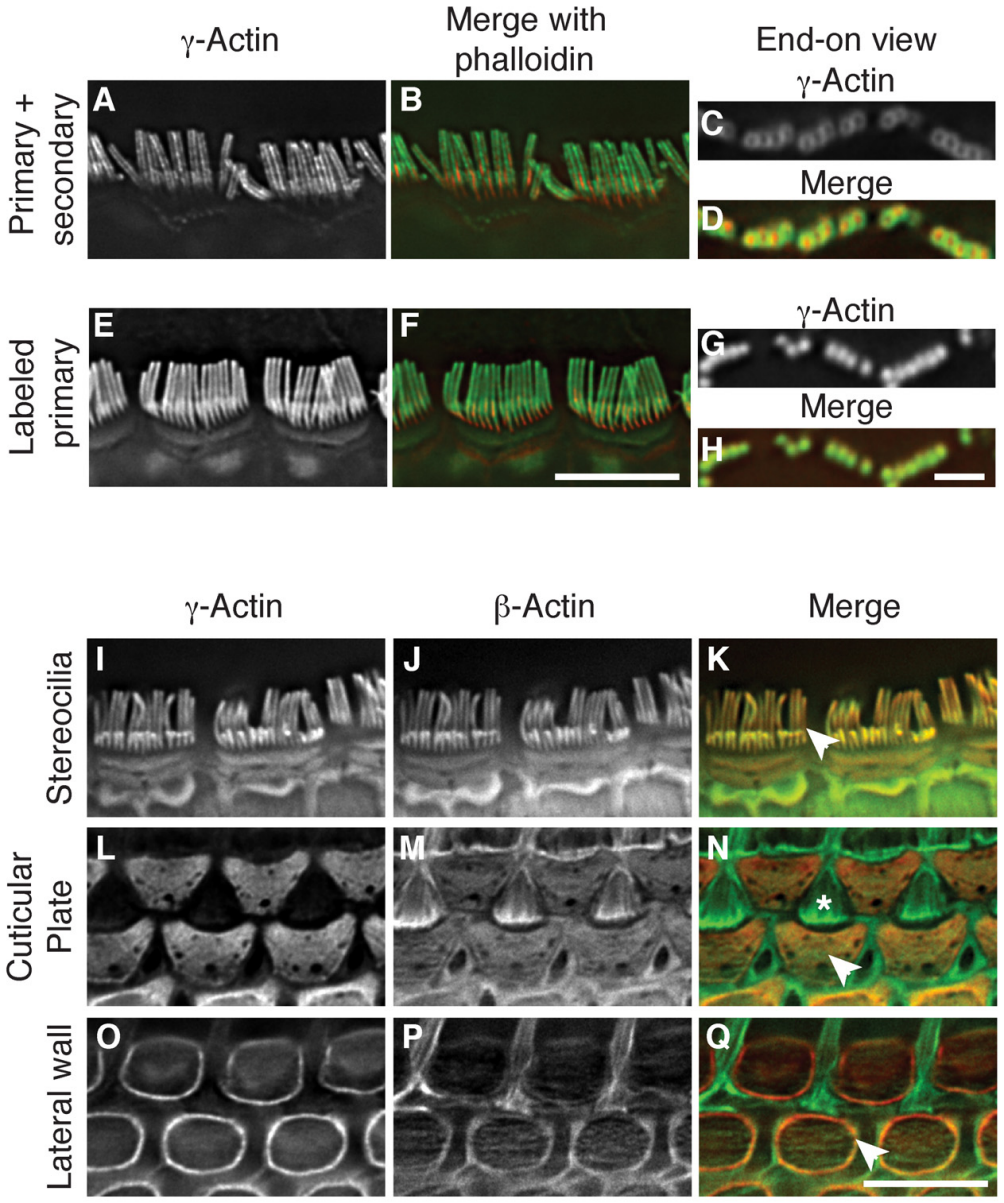
β-actin and γ-actin colocalize in hair cells. (A–H) Comparison of γ-actin localization using unlabeled anti-γ-actin antibody with a fluorescent secondary antibody (A–D) or using the same anti-γ-actin antibody coupled directly to a fluorescent dye (E–H). Detection with a secondary antibody results in a peripheral localization pattern when viewed laterally (A–B) or end-on (C–D). Dye-conjugated anti-γ-actin antibody labels stereocilia uniformly and completely overlays with phalloidin in lateral (E–F) or end-on (G–H) views. In merged images, γ-actin is green and phalloidin is red. (I–Q) Localization of β-actin and γ-actin using primary antibodies conjugated to two different fluorescent dyes. γ-Actin and β-actin colocalize in stereocilia (I–K), cuticular plate (L–N) and lateral wall (O–Q). In merged images, β-actin is green and γ-actin is red. Arrowheads indicate the structure of interest and the asterisk in (N) marks the top of a support cell that borders outer hair cells. Bars in F and Q, 5 µm; H, 2 µm.

Using paraformaldehyde-fixed tissue that was post-fixed in methanol, we found that a monoclonal anti-γ-actin primary antibody in combination with a secondary antibody gave a previously described peripheral localization pattern (Figure 2A–2D) [9]. In contrast, the same anti-γ-actin antibody directly coupled to a fluorescent dye was observed to label stereocilia uniformly (Figure 2E–2H). Moreover, unconjugated β-actin antibodies detected with a secondary antibody produced the same peripheral localization pattern as observed with unlabeled antibodies to γ-actin (Figure S2). Finally, β-actin and γ-actin appeared to colocalize in cells simultaneously labeled with dye-conjugated antibodies to each isoform (Figure 2I and 2K). Therefore, at the level of light microscopy, we did not detect differential localization of β-actin and γ-actin in IHC stereocilia. These data further suggest that secondary antibodies are either unable to fully penetrate densely packed stereocilia actin, or are sterically prevented from binding their primary antibody targets.

Hair cells have other actin-rich structures including the cuticular plate, which is an actin meshwork where stereocilia are anchored, and the lateral wall, which features an actin-spectrin lattice. In agreement with previous studies [18], [29], we found both isoforms in the cuticular plate (Figure 2L–2N) and in the lateral wall, where γ-actin appears to be more abundant (Figure 2O–2Q). Although the ratio of β-actin to γ-actin may vary, our immunofluorescent studies did not identify any structure in hair cells that contained only a single cytoplasmic actin isoform.

### Stereocilia formation requires cytoplasmic actin

**Figure 3:**
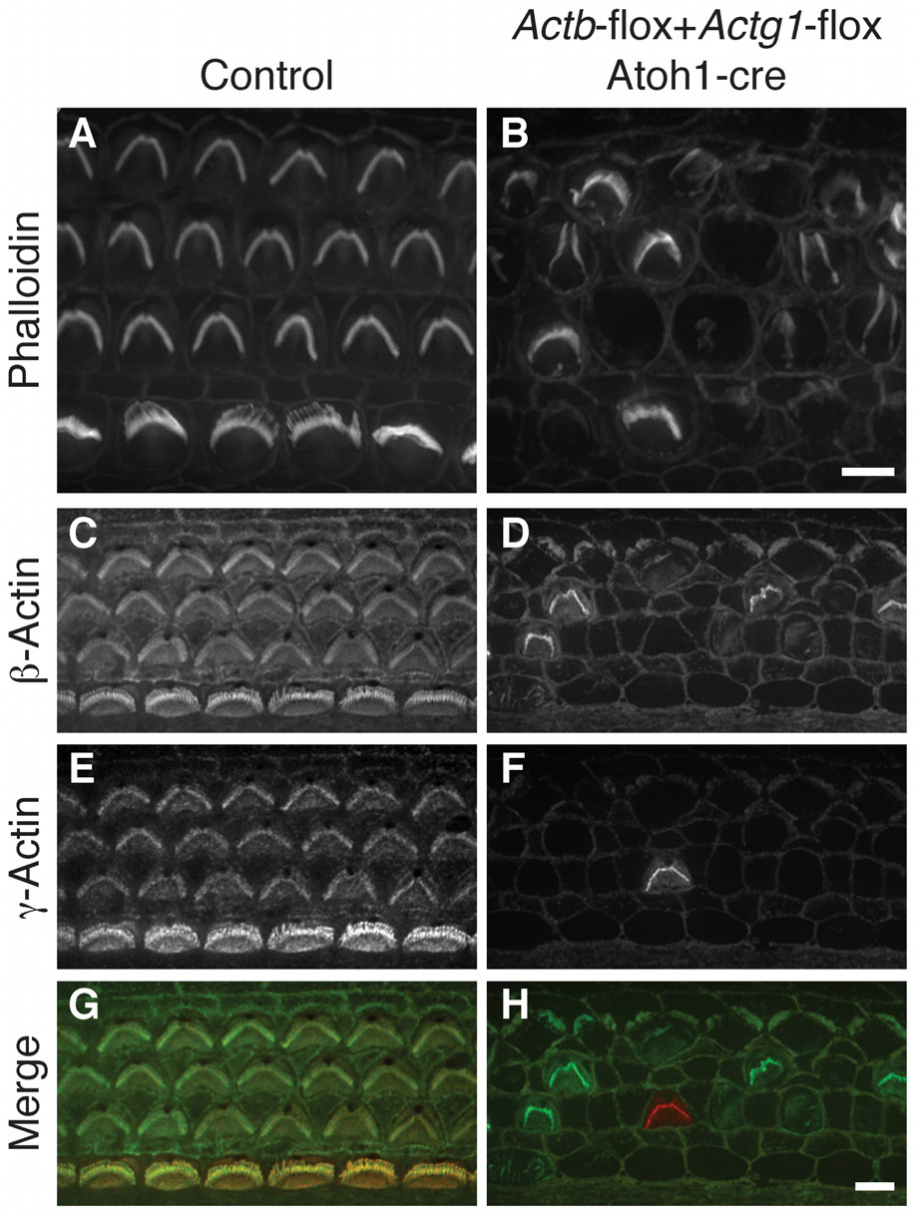
*Actb Actg1* double knockout cells do not develop stereocilia. (A, B) Phalloidin staining of organ of Corti from control (A) or *Actb*-flox *Actg1*-flox *Atoh1*-cre (double knockout) (B) P5 pups shows normal stereocilia in control but generally absent stereocilia in the double knockout. (C–H) double label with dye-conjugated antibodies to β-actin or γ-actin show that remaining stereocilia contain one of the actin isoforms. In merged images in (G–H) β-actin is green and γ-actin is red. Bars, 5 µm.

The finding that neither cytoplasmic actin is individually essential for stereocilia formation raised the possibility that cytoplasmic actins are entirely dispensable for auditory development. Therefore, we generated *Actb Actg1 Atoh1*-cre mediated double knockout hair cells. In P5 mice, double knockout hair cells were largely devoid of phalloidin-stained stereocilia (Figure 3A and 3B). The few remaining bundles immunostained for either β-actin or γ-actin (Figure 3C–3H) and likely persisted because of incomplete cre-mediated recombination at this age. We conclude that either β-actin or γ-actin is required for stereocilia development even though each is dispensable, suggesting that other members of the actin family are unable to compensate for loss of cytoplasmic actin function in hair cells.

### *Actb* and *Actg1* hair cell knockout mice develop distinct patterns of progressive hearing loss

Because of the ubiquitous expression pattern of cytoplasmic actins, aspects of the slowly progressive hearing loss recently observed in whole-body *Actg1^−/−^* mice may be unrelated to hair cell or stereocilia function [9]. Therefore, we measured auditory brainstem response (ABR) in *Actg1*-flox *Atoh1*-cre mice, where cre expression in the auditory system is limited to inner hair cells, outer hair cells and spiral ganglion cells [27]. Correspondingly, in the organ of Corti, we observed loss of γ-actin in both inner and outer hair cells (Figure S3). ABR uses subdural electrodes to record synchronous neural activity from an anesthetized mouse in response to a short tone burst of a given frequency and sound level. The lowest sound intensity level that produces a neural response is the hearing threshold.

**Figure 4:**
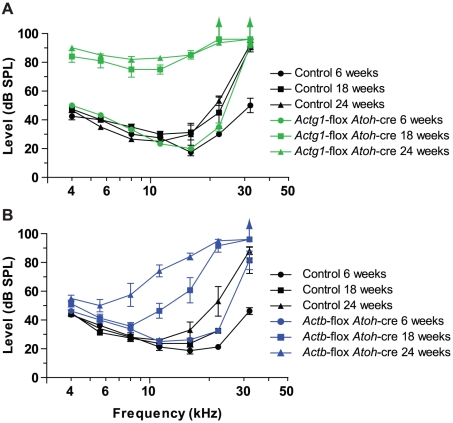
Mice with hair cells deficient for either β-actin or γ-actin develop distinct forms of progressive hearing loss. Auditory brainstem response (ABR) thresholds at 6, 18 and 24 weeks of age. (A) *Actg1*-flox *Atoh1*-cre mice have near normal ABR thresholds for sound tones between 4 and 22 kHz at 6 weeks of age, but elevated thresholds at all frequencies at 18 and 24 weeks of age. (B) *Actb*-flox-*Atoh1*-cre mice have progressive loss of hearing starting with higher frequency sounds.

Similar to *Actg1^−/−^* mice, we found that 6 week-old *Actg1*-flox *Atoh1*-cre mice had thresholds that were indistinguishable from control mice at frequencies between 4 kHz and 22 kHz, while the 32 kHz threshold was elevated (Figure 4A). However, by 18 weeks-of-age, *Actg1*-flox *Atoh1*-cre mice had significantly elevated thresholds at all frequencies tested (Figure 4A). Although this pattern of hearing loss corresponds very well with that observed for *Actg1^−/−^* mice of the same age [9], differences between the two models were apparent at later stages. While 24 week-old *Actg1*-floxed *Atoh1*-cre mice had thresholds at 22 kHz and 32 kHz that were above 90 dB SPL, the maximum sound level tested, hearing between 4 and 16 kHz was not markedly worse than at 18 weeks-of-age (Figure 4A). In contrast, hearing function worsened in *Actg1^−/−^* mice over the same interval [9], suggesting additional functions for γ-actin outside of hair cells during this time frame. Together, these data demonstrate that *Actg1*-flox *Atoh1*-cre mice have nearly normal hearing as young adults, but develop progressive loss of hearing at all frequencies. The early stages of hearing loss recapitulate that of *Actg1^−/−^* mice and suggest critical hair-cell specific functions for γ-actin.

*Actb*-flox *Atoh1*-cre mice provide an excellent system for comparing β-actin function to γ-actin function in hair cells. In these mice at 6 weeks-of-age, ABR thresholds between 4 kHz and 16 kHz were similar to control littermates, while the 22 kHz threshold was slightly elevated and the 32 kHz threshold was markedly elevated (Figure 4B). By 18 weeks-of-age, hearing loss had progressed to include drastic elevation of the 22 and 32 kHz thresholds and moderate elevation of 11 and 16 kHz thresholds. These thresholds were further elevated at 24 weeks-of-age. In addition, lower frequencies, most prominently 8 kHz, were also higher than in control animals (Figure 4B).

Therefore the loss of β-actin preferentially affects high frequency hearing while the loss of γ-actin affects all frequencies more uniformly and progresses more rapidly. These different patterns of hearing loss suggest that β-actin and γ-actin have different and non-redundant functions in hair cells.

### β-actin and γ-actin deficient stereocilia have distinct pathology

**Figure 5:**
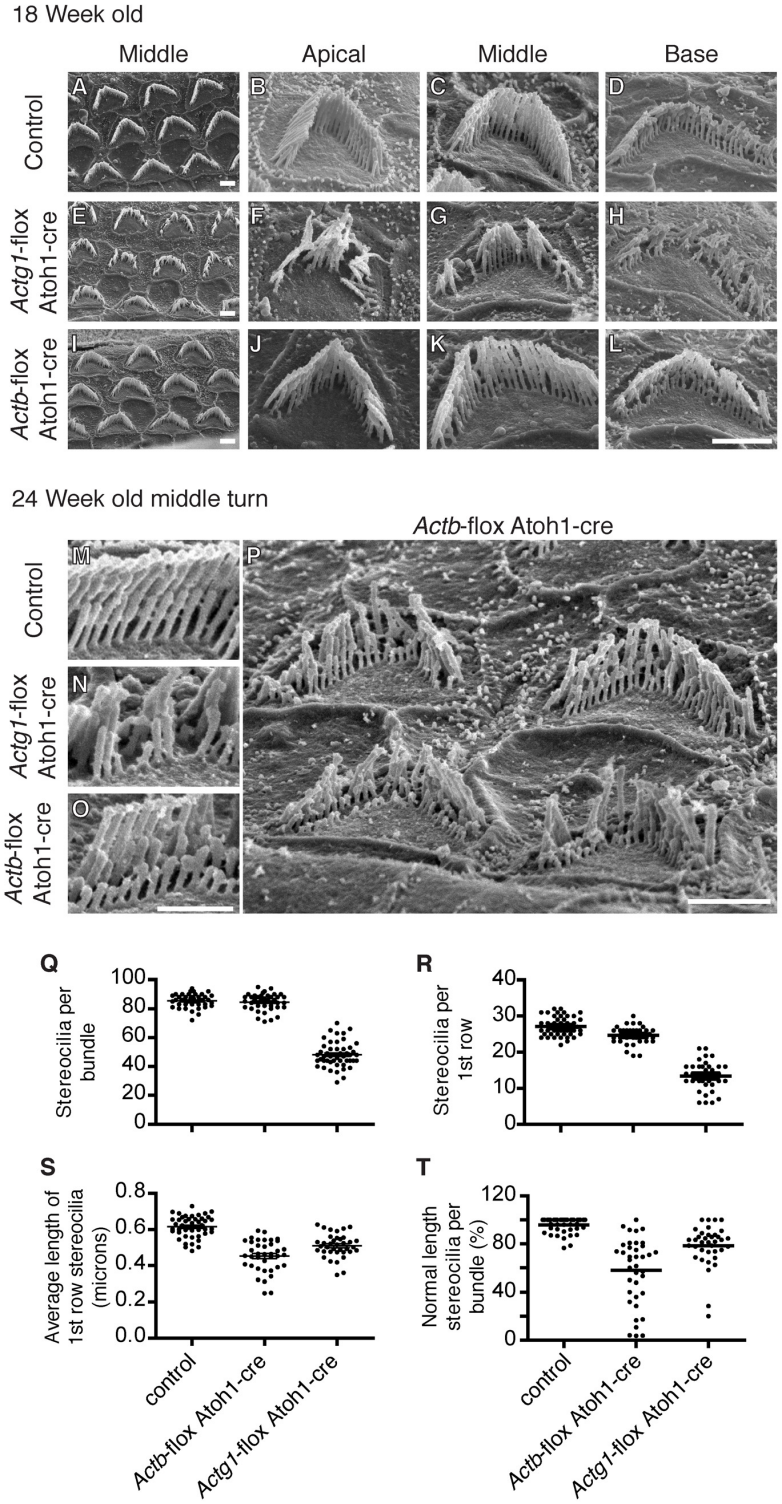
β-actin and γ-actin deficient stereocilia develop distinct pathology. Analysis of stereocilia morphology by scanning electron microscopy at 18 weeks-of-age (A–L) and 24 weeks-of-age (M–P). (A–D) 18 Week-old control stereocilia. (E–H) 18 Week-old γ-actin deficient stereocilia from all cochlear locations are degraded with significant numbers of individual stereocilia missing (Q). (I–L) β-Actin deficient stereocilia at 18 weeks of age from the apical (J) and middle turns (I,K) are similar to control, while stereocilia from the basal turn are abnormally short (L). Bars, 2 µm. (M–O) Comparison of stereocilia pathology at 24 weeks-of-age. γ-Actin deficient bundles are missing individual stereocilia while β-actin deficient stereocilia are uniformly shortened. Bar, 1 µm. (P) β-Actin deficient stereocilia from the middle turn have developed pathology ranging from mild to severe shortening of most members of whole rows in the bundle. Bars, 2 µm. (Q) Number of stereocilia in 18 week-old hair cell bundles, only *Actg1*-flox *Atoh1*-cre stereocilia numbers are statistically different. 40 OHC bundles from 4 ears of each genotype were analyzed. (R–T) Analysis of the first row of stereocilia of bundles from 24 week-old mice. The number (R) and average length (S) of first row stereocilia were determined. The percent of these stereocilia that were of normal length (within 2 standard deviations of the control average) was calculated (T). In (R–T), each dot plotted represents one cell (more than 35 cells from 3 mice of each genotype) and the bar is the mean. All groups are statistically different from all other groups.

Different frequencies of sound are detected at discrete locations along the length of the cochlea, with the highest frequencies detected at the basal end and the lowest frequencies at the apical end. Since hearing loss in mice with γ-actin or β-actin deficient hair cells is frequency dependent, we examined the morphology of OHC stereocilia from apical, middle and basal regions. At 18 and 24 weeks-of-age, hair bundles from all regions of *Actg1*-flox *Atoh1*-cre cochlea were degraded with a significant loss of individual stereocilia (Figure 5E–5H, 5N, 5Q). Other stereocilia in the same bundle were shortened, but many retained apparently normal morphology (Figure 5N). The consistent stereocilia pathology in apical, middle and basal regions corresponds with the uniform elevation of ABR thresholds across the frequency range (Figure 4A). In contrast, hair bundles from *Actb*-flox *Atoh1*-cre mice had less severe morphological defects at 18 weeks-of-age (Figure 5I–5L, 5Q). In keeping with normal ABR thresholds at low and middle frequencies, we did not observe obvious stereocilia defects in the apical or middle regions (Figure 5I–5K). Outer hair cells from the basal region, which detects high frequency sounds, had an altered morphology characterized by shortened stereocilia with heights that were variable and not in register with neighboring stereocilia (Figure 5L).

At 24 weeks-of-age, pathology of β-actin-deficient stereocilia progressed, now affecting stereocilia from the middle cochlear turn, corresponding with the progression of hearing loss in *Actb*-flox *Atoh1*-cre mice (Figure 5O–5P). β-Actin-deficient stereocilia lengths varied considerably from cell to cell. In some cases, stereocilia were of normal length but lacked the precise height registration with neighboring stereocilia that is a hallmark of normal morphology. In other cases individual stereocilia and whole rows of stereocilia were severely shortened (Figure 5O–5P). Nevertheless, β-actin-deficient and γ-actin-deficient stereocilia had distinct morphologies (Figure 5M–5O).

To compare β-actin deficient stereocilia to γ-actin deficient stereocilia, we quantified morphological changes in 24 week-old stereocilia by measuring the lengths of the first (shortest) row of stereocilia in bundles from all rows of OHCs in the middle region of the cochlea. Consistent with our previous observations, γ-actin-deficient hair cells had markedly fewer stereocilia (Figure 5R) that had a shorter average length than control stereocilia (Figure 5S). In contrast, β-actin deficient hair cells had only slightly fewer stereocilia than control cells (Figure 5R), and these stereocilia were shorter than either control or γ-actin deficient stereocilia (Figure 5S). The difference in average length between β-actin and γ-actin deficient stereocilia was statistically significant but appeared slight, perhaps due to a few very short γ-actin-deficient stereocilia mixed in with remaining members of normal height (Figure 5N). Therefore, we also compared the percentage of stereocilia that were of normal height, defined here as measured heights within two standard deviations of the average length in controls. Using this criterion, we found that appreciably more γ-actin deficient bundles than β-actin deficient bundles retained a high fraction of normal length stereocilia (Figure 5T). This data demonstrates that γ-actin deficient hair cells have lost a large percentage of stereocilia, with most remaining stereocilia retaining normal length. In contrast, β-actin deficient stereocilia are largely retained but are more consistently shortened in length.

### Hearing loss and stereocilia pathology depend on γ-actin concentration

**Figure 6:**
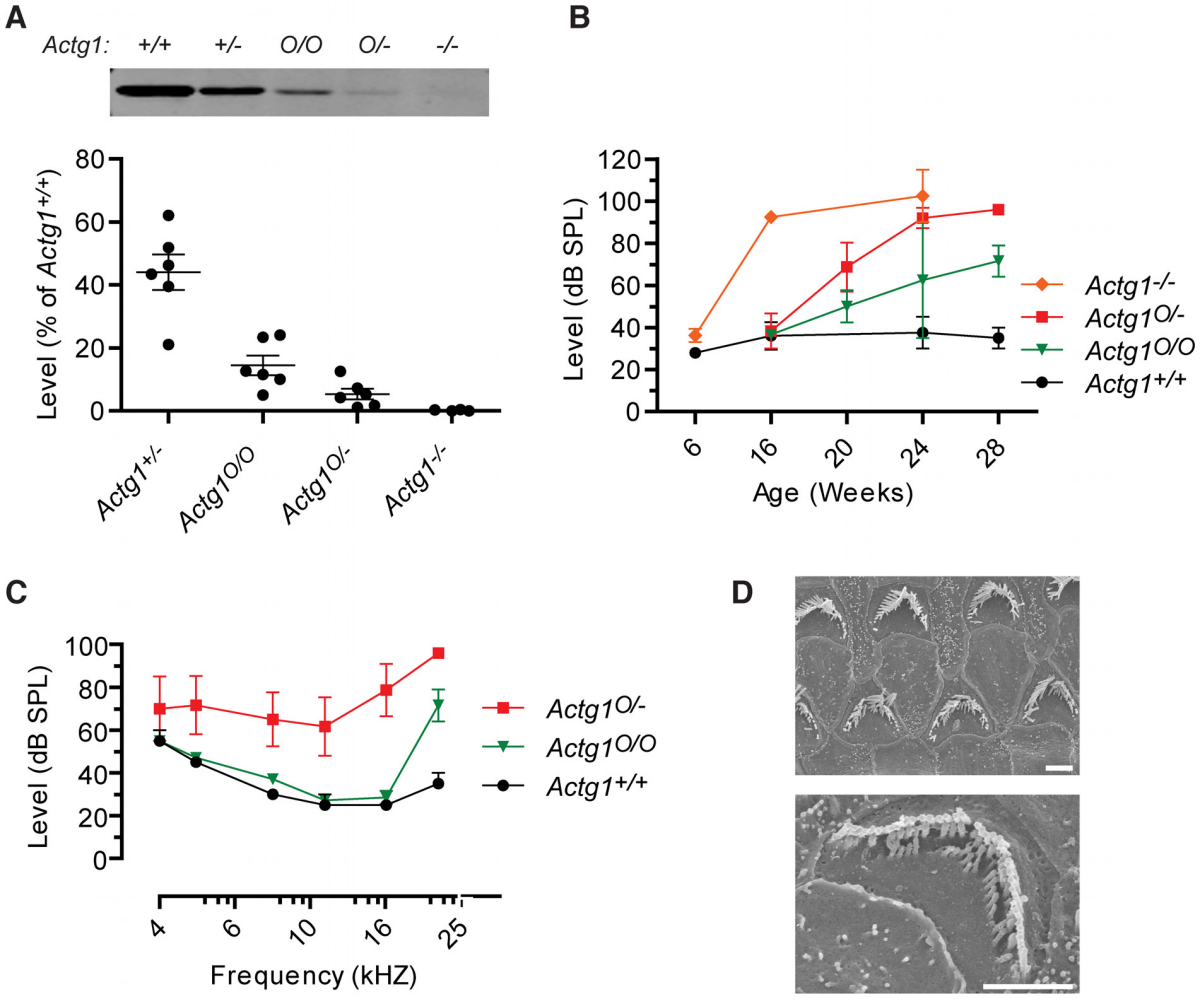
Onset and progression of hearing loss depends on γ-actin concentration. (A) γ-actin protein levels in cochlea from *Actg1^+/+^*, *Actg1^+/−^*, *Actg1^O/O^*, *Actg1^O/−^* and *Actg1^−/−^* mice were quantified using immunoblot analysis. (B) 22 kHz auditory brainstem response (ABR) thresholds were determined at the indicated ages, demonstrating that the onset and rate of hearing loss progression depend on the concentration of γ-actin. *Actg1^−/−^* data is replotted from [9]. (C) ABR thresholds from 28 week-old *Actg1^+/+^*, *Actg1^O/O^* and *Actg1^O/−^* mice. (D) Scanning electron microscopy of outer hair cells from *Actg1^O/−^* mice, demonstrating that mice with hypomorphic γ-actin expression develop a phenotype similar to that of hair cells devoid of γ-actin. Bars, 2 um.

β-actin and γ-actin-deficient stereocilia develop different phenotypes, suggesting that each actin isoform has a distinct role in maintaining stereocilia morphology and auditory function. We reasoned that the severity of the phenotype should depend on cellular concentration of each isoform. We generated a series of mice with variable levels of γ-actin protein by taking advantage of a hypomorphic allele (*Actg1* ^O^) caused by insertion of a neomycin cassette into intron 1 [30]. Based on quantitative immunoblot analysis of cochlear extracts, we determined that *Actg1* ^+/−^, *Actg1* ^O/O^ and *Actg1* ^O/−^ mice express 44%, 15% and 5%, respectively, of wild-type γ-actin levels (Figure 6A). We previously found that *Actg1* ^+/+^ and *Actg1* ^+/−^ mice had similar ABR thresholds [9]. Here, we found that hearing loss onset and progression depend on the concentration of γ-actin. *Actg1* ^O/O^ and *Actg1* ^O/−^ mice both had a normal 22 kHz ABR threshold at 16 weeks-of-age, a time point at which *Actg1^−/−^* mice have profound hearing loss (Figure 6B) [9]. At subsequent time points, both lines progressively lost hearing sensitivity, with the lower γ-actin level in *Actg1* ^O/−^ mice correlating with a faster rate of hearing loss (Figure 6B). By 28 weeks-of-age, *Actg1* ^O/−^ mice had marked hearing loss across the range of tested frequencies, recapitulating the pattern of impairment observed in *Actg1*-flox *Atoh1*-cre mice (Figure 6C). Finally, SEM analysis demonstrated that *Actg1* ^O/−^ stereocilia morphology at 28 weeks phenocopied the pathology observed in γ-actin-deficient hair cells, where in both cases hair bundles appear degraded with individual stereocilia missing (Figure 6D). Therefore, these distinct phenotypes depend on the γ-actin concentration, further suggesting that γ-actin has a specific cellular function.

### Progressive hearing loss in *Actg1* hair cell knockout mice is not rescued by cadherin23

**Figure 7:**
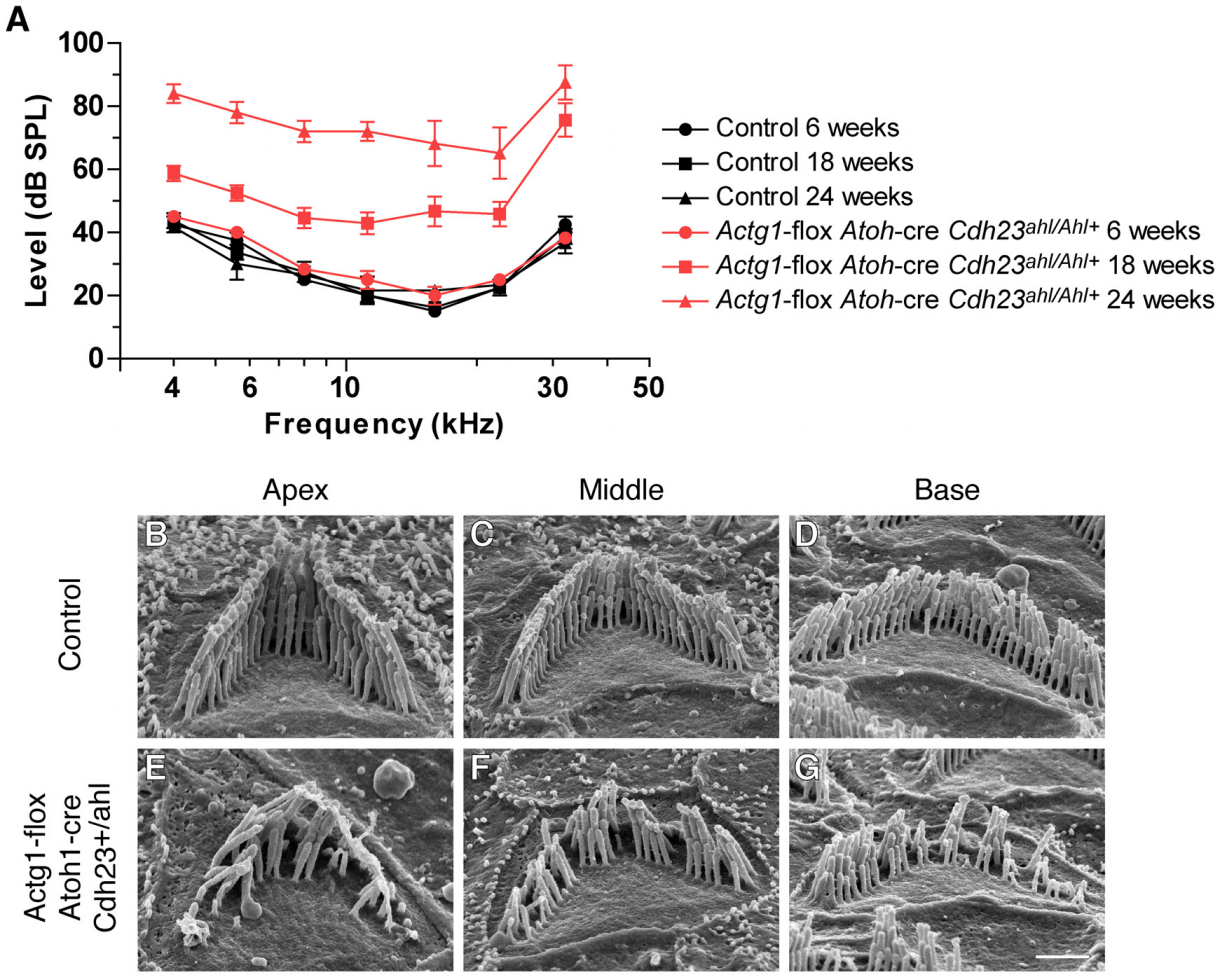
*Cdh23^Ahl+^* does not rescue progressive hearing loss in mice with γ-actin–deficient hair cells. (A) *Actg1-*flox *Atoh1*-cre *Cdh^Ahl+/ahl^* mice have elevated ABR thresholds at 18 and 24 weeks of age. (B–G) SEM analysis of stereocilia morphology at 18 weeks of age. OHC hair bundles from control *Actg1-*flox *Cdh^Ahl+/ahl^* mice (B–D) have normal stereocilia morphology while OHC hair bundles from *Actg1-*flox *Atoh1*-cre *Cdh^Ahl+/ahl^* mice have a degraded appearance with missing individual stereocilia. Bar is 1 µm.

The *Actg1*-floxed *Atoh1*-cre mice were characterized on the C57Bl/6 inbred genetic background, which is predisposed to progressive hearing loss by the age-related hearing loss (AHL) susceptible allele of the cadherin23 gene (*Cdh23^ahl^*) [25]. *Cdh23^ahl^* transcripts lack exon 7, while the dominant-acting resistance allele, *Cdh^Ahl+^*, encodes the full-length transcript and protects against AHL [24], [25]. Since cadherin23-mediated tip link stability might impact stereocilia maintenance, we next determined if the hearing loss and stereocilia pathology that we observed in *Actg1*-floxed *Atoh1*-cre mice depends on the *Cdh^ahl^* allele. We crossed mice harboring *Cdh23^Ahl+^* on an otherwise C57Bl/6 background [24] to *Actg1*-floxed *Atoh1*-cre mice. At six weeks of age, *Actg1*-floxed *Atoh1*-cre *Cdh23^Ahl+/ahl^* mice had ABR thresholds similar to control *Actg1*-floxed *Cdh23^Ahl+/ahl^* mice. However, ABR thresholds in these γ-actin-deficient mice were markedly increased at 18 weeks of age at all frequencies tested, and were further elevated at 24 weeks of age (Figure 7A). As expected, both control and *Actg1*-floxed *Atoh1*-cre mice carrying *Cdh23^Ahl+^* had lower ABR thresholds at high frequencies (Figure 7A) compared to mice on a homozygous *Cdh23^ahl/ahl^* background (Figure 4A). In addition, *Cdh23^AHL+^* generally slowed the progression of hearing loss in *Actg1*-floxed *Atoh1*-cre mice (Figure 7A). Nevertheless, SEM analysis of OHC stereocilia from 18 week old *Actg1*-floxed *Atoh1*-cre *Cdh23^Ahl+/ahl^* mice revealed the characteristic γ-actin-deficient pathology, including hair bundle degradation with loss of individual stereocilia (Figure 7B–7G). Together, these data demonstrate that progressive hearing loss and stereocilia degradation associated with γ-actin deficiency occurs regardless of defects in Cdh23.

## Discussion

Mice with β-actin or γ-actin deficient auditory hair cells form functional, morphologically normal stereocilia and have normal hearing at young ages. However, when both isoforms are ablated, stereocilia are absent. Together, these data demonstrate that cytoplasmic actin is required during hair cell development, but that β-actin and γ-actin are redundant. Following development and the onset of hearing, hair cells and their stereocilia must be maintained for the life of the organism. During this phase, β-actin and γ-actin are both required because each knockout develops distinct stereocilia pathology and each has a distinct pattern of progressive hearing loss.

### *Actb* and *Actg1* gene products, rather than gene regulation, are important for stereocilia maintenance

The mammalian cytoplasmic actins are 99% identical; consequently, it has been unclear whether the proteins have different functions. Instead, two actin genes might be necessary to properly regulate the level of cellular actin, as is the case in *Drosophila*. Flies have two essential, closely related cytoplasmic actin genes. Lethality is rescued by expressing only one actin protein sequence from both promoters, demonstrating that gene regulation, rather than protein function, is the critical factor [31]. The analogous experiment has not been done in any mammalian system, but several lines of evidence suggest that gene regulation is not a limiting factor in hair cells. First, both isoactins can be expressed in hair cells at sufficient levels to independently support normal development. Second, knockout of *Actg1* does not change the level of total actin in the cochlea because other isoforms are upregulated [9], arguing that changes in the composition, but not the concentration, of actin results in the observed pathology. Finally, *Actb* or *Actg1* ablation results in different phenotypes. Furthermore, varying the dose of γ-actin changes the age of onset and the rate of progression of hearing loss, but results in the same end γ-actin-specific phenotype. These relationships strongly imply that each cytoplasmic actin gene product has distinct cellular functions.

*Actb* transcripts have a “zipcode” sequence element in their 3′UTR that mediates interactions with binding proteins that both regulate expression and transport the transcript to specific regions of the cell, such as neuronal growth cones or the leading edge of fibroblasts [32]–[34]. γ-Actin mRNA lack the zipcode sequence, and resulting differences in transcript localization and regulation likely contribute to the evolutionary conservation of both cytoplasmic actin genes. The zipcode sequence has not been studied in hair cells. Although β-actin and γ-actin seem to be colocalized in hair cells, transcript localization may nevertheless contribute to phenotypic differences seen in this study.

### β-actin and γ-actin colocalize in hair cells but knockouts have distinct phenotypes

Differential subcellular localization of β-actin and γ-actin in hair cells could indicate that each protein has distinct functions and explain why each knockout phenotype is different. However, we detected both actin isoforms colocalized in inner hair cell stereocilia, cuticular plate and lateral wall. γ-Actin may be more abundant than β-actin in the cuticular plate [18] and more strikingly so in the lateral wall (Figure 2O–2P) [18]. Therefore, loss of γ-actin may affect these structures more than the loss of β-actin if the local actin concentration is not maintained.

Alternatively, colocalized and copolymerized β-actin and γ-actin may have distinct functions based on unique biochemical properties of each isoform, an argument bolstered by a recent biochemical study of recombinant γ-actin and β-actin [3]. γ-Actin, particularly in the calcium bound form, was shown to polymerize, depolymerize and treadmill significantly more slowly than β-actin. In addition, γ-actin and β-actin readily copolymerize, supporting our immunofluorescence data suggesting that stereocilia actin filaments are copolymers of both isoforms. Critically, the polymerization properties of the copolymer reflect the ratio of β-actin to γ-actin [3]. In this way, ablating or altering the concentration of one actin isoform in stereocilia likely alters the biochemical properties of the actin array.

We observed differences in the length of β-actin-deficient stereocilia and the stability γ-actin-deficient stereocilia, which could be explained in part by different polymerization kinetics of the actin isoforms. Stereocilia actin undergoes continuous treadmilling, with new monomers adding to actin filaments at stereocilia tips and depolymerizing at the stereocilia base [35]. Critically, the rate of actin treadmilling is precisely coupled to stereocilia length, suggesting that actin polymerization must be tightly regulated in order to maintain the proper hair bundle architecture [35]. Changes in the concentration of β-actin and γ-actin may have more significant consequences in hair cells than in other tissues. As Rubenstein and colleagues point out, the uniquely high calcium concentration in stereocilia may result in calcium-bound β-actin and γ-actin, which have dramatically different polymerization properties than the more commonly found magnesium-bound species [3]. Since stereocilia must be maintained for the life of the organism, it is conceivable that changes in the actin polymerization rate could account for the observed, slowly developing morphological defects.

We found that stereocilia develop normally in β-actin or γ-actin-deficient hair cells. Therefore, if actin isoform dependent polymerization kinetics contributes to the pathology we observed, then actin dynamics must be different in developing stereocilia and adult stereocilia. The rate of actin treadmilling in stereocilia has thus far only been measured in perinatal hair cell explants, leaving open the possibility that the treadmilling rate may be significantly different in adult stereocilia. Such differences may explain why β-actin or γ-actin deficient stereocilia develop normally but deteriorate in adults.

In addition to changes in polymerization properties, β-actin and γ-actin may have different affinities for a subset of actin binding proteins. Indeed, the excellent specificity of actin isoform specific antibodies suggests that each protein can be distinguished on the basis of protein-protein interactions. In keeping with this, cofilin [36], ezrin [37], l-plastin [38], βCAP73 [39], Thymosin b4 [40] and profilin [41] differentially interact with cytoplasmic and muscle actin isoforms while Annexin V is reported to bind γ-actin, but not β-actin [42]. Recent advances in recombinant cytoplasmic actin expression and more sophisticated assays of biochemical function should facilitate the discovery of additional isoform specific binding proteins.

### Cytoplasmic actins in progressive hearing loss

Progressive hearing loss is common in the aging human population, resulting from a combination of environmental and genetic factors. Genetic mapping studies using inbred mouse lines have identified several genes that predispose mice for age-related hearing loss (AHL), including *Cdh23^ahl^* and *ahl8*, which encodes the R109H variant of fascin2 [25], [43], [44]. Assembling the products of genes that promote AHL into molecular pathways may provide useful insight into maintenance of auditory function. Along these lines, fascin2 and γ-actin are likely connected because hair cells that are γ-actin-deficient or carry fascin2 R109H develop phenotypically similar stereocilia degradation [44]. Furthermore, Johnson and colleagues have recently noted that in mice the respective genes (*Actg1* and *Fscn2*) are separated by only 13kb and that transcript expression is coordinately regulated [44]. However, in contrast to the fascin2 R109H phenotype [43], progressive hearing loss due to γ-actin-deficiency does not require homozygosity for *Cdh23^ahl^*. Therefore, γ-actin likely also functions in pathways distinct from cadherin23 and fascin2 that are important for hair cell maintenance. This is consistent with the finding that γ-actin is required for normal growth and viability of isolated fibroblasts [45]. Finally, since mouse models lacking either β-actin or γ-actin develop distinct forms of progressive hearing loss, we conclude that the unique properties of each cytoplasmic actin are necessary to maintain proper structure and function of long-lived auditory hair cells.

## Materials and Methods

### Ethics statement

The experimental protocols in this study were reviewed and approved by the University of Minnesota Institutional Animal Care and Use Committee.

### Generation of the *Actb^flox^* allele

A targeting vector containing loxP sites flanking exons 2 and 3, as well as a neomycin cassette, was electroporated into 129S6 murine embryonic stem cells at the Gene Targeting Mouse Service Core (University of Cincinnati, Cincinnati, OH). A clone with successful recombination was identified by Southern blot analysis, karyotyped and injected into C57Bl/6 blastocysts at the University of Wisconsin-Madison (Figure S1B). Chimeric males were bred to C57Bl/6 females to determine germline transmission of the floxed allele. The neomycin cassette was removed by crossing to a line expressing EIIa-cre [46] as described [30], generating *Actb^flox^* mice, which were backcrossed to C57Bl/6 for 10 generations. Details of the vector construction, Southern blot and genotyping procedures can be found in Text S1.

### Immunofluorescent microscopy

Adult mice were perfused with 4% paraformaldehyde (PFA) in PBS, cochlea were dissected, additional fixative was gently perfused through the round and oval windows and then incubated in the same fixative solution for 2 hours at room temperature. Cochlea were washed in PBS and then decalcified in 170 mM EDTA in PBS at 4°C for 16 hours. Cochlea from post-natal mice at the indicated ages, where P0 is the day of birth, were dissected and immersed in fixative for 16 hours at 4°C and were not decalcified. The organ of Corti was dissected, postfixed in 100% methanol at −20°C for 10 minutes, rinsed in PBS and permeablized in 0.5% triton X-100 in PBS for 20 minutes at room temperature. Tissue was blocked for 1 hour in 5% goat serum in PBS prior to incubation with the indicated antibodies. Samples were mounted in ProLong anti-fade reagent and viewed on a Deltavision PersonalDV microscope equipped with a 100×1.4 NA objective (Applied Precision). Stacks of images were collected at 0.20 µm intervals and subsequently deconvolved using Resolve3d software (Applied Precision).

### Scanning electron microscopy

Cochlea were fixed in 2.5% glutaraldehyde in 0.1 M sodium cacodylate buffer with 1 mM CaCl_2_ by perfusing dissected cochlea through the round and oval windows followed by incubation in the same solution at room temperature for 4 hours. Following decalcification in 170 mM EDTA at 4°C for 16 hours, the organ of Corti was dissected and processed for SEM as described [9]. Briefly, tissue was successively incubated in 2% each arginine, glycine, glutamic acid and sucrose in water, 2% each tannic acid and guanidine-HCl in water and then 1% osmium tetroxide. Samples were critical point dried from CO_2_ and sputter coated with platinum before viewing on a cold field emission scanning electron microscope (Hitachi S4700). Stereocilia were measured using ImageJ software and statistical analysis (one-way ANOVA with Tukey post-test) was performed using GraphPad Prism software.

### Mice

The *Actg1^flox^* alleles, mice expressing cre recombinase from either the *Foxg1* locus (Jackson labs, on the C57Bl/6 background, stock number 006084 [26]) or from a *Atoh1*-cre transgene [27], and mice carrying the *Cdh23^Ahl+^* allele on an otherwise C57Bl/6 background [24] (Jackson labs, stock number 002756) have been previously described. Standard mouse husbandry practices were used to generate the indicated lines. *Actb*-flox *Atoh1*-cre and *Actg1*-flox *Atoh1*-cre mice were backcrossed to C57Bl/6 mice for 5 generations. Animals were housed and treated in accordance with the standards set by the University of Minnesota Institutional Animal Care and Use Committee.

### Antibodies

Monoclonal mouse anti-γ-actin antibody clone 1–37 [47] IgG was purified from ascites using a T-gel Purification kit (Pierce) and conjugated to either Alexa-488 or Alexa-568 fluorescent dyes using a Monoclonal Antibody Labeling Kit (Invitrogen) following the manufacturer's instructions. Unlabeled antibodies were detected with goat anti-mouse secondary antibodies labeled with Alexa-488 (Invitrogen). Unlabeled (clone AC-74) and FITC labeled anti-β-actin antibodies (clone AC-15) was obtained from Abcam.

### Auditory Brainstem Response (ABR)

ABR waveforms were collected as previously described [9] for frequencies between 4 kHz and 32 kHz at half-octave intervals, starting at supra-threshold levels and decreasing in 5 dB steps to a sub-threshold level. A Tucker-Davis Technologies System 3 was used to generate symmetrically shaped tone bursts 1 ms in duration with 300 µs raised cosine ramps that were delivered to a calibrated magnetic speaker. Mice were anesthetized with Avertin and scalp potentials were recorded with subdermal electrodes with signals amplified 20,000 times, bandpass filtered between 0.03 and 10 kHz, digitized using a 20,000 kHz sampling rate and subjected to artifact rejection. Stacked waveforms were compared and the lowest level of stimulation that evoked an unambiguous ABR waveform was designated as the threshold.

### Immunoblot analysis

As previously described [9], cochlea were dissected from mice of the indicated genotypes, frozen in liquid nitrogen, ground into powder, boiled in 1% SDS buffer and centrifuged to remove insoluble material. Protein concentration in the resulting lysate was determined by A_280_ measurement. Equal amounts of protein were separated by SDS-PAGE, transferred to nitrocellulose membranes and probed with anti-γ-actin antibody pAb7577 [47]. Fluorescently labeled secondary antibodies were detected and quantified using an Odyssey infrared scanner and software (Li-Cor Biosciences).

## Supporting Information

Figure S1 Generation of the Actb^flox^ allele (A) Targeting scheme used to “flox” exons 2 and 3 of the Actb locus and subsequent Cre-mediated recombined alleles. Gray boxes denote exons and loxP sites are depicted as triangles. Abbreviations: EcoRI, E; HindIII, H; BamHI, B. (B) Southern blots of HindIII digested ESC DNA hybridized with the appropriate probe. The WT allele yielded an ∼15 kb fragment, while the targeted alleles resulted in ∼6 kb (5′ probe) and 9.4 kb (3′ probe) fragments.
(2.21 MB EPS)

Figure S2 Anti-β-actin antibody detected with secondary antibodies stains stereocilia periphery. Organ of Corti from wild-type mice was immunostained with primary anti-β-actin antibody AC-74 detected with a fluorescent secondary antibody. β-Actin staining is enriched at the periphery of stereocilia compared to the phalloidin stained core.
(1.23 MB EPS)

Figure S3 Hair cells from *Actg1*-flox *Atoh1*-cre mice are γ-actin deficient. The organ of Corti isolated from adult *Actg1*-flox *Atoh1*-cre mice or control mice were stained with anti-γ-actin antibody. γ-Actin is largely undetectable in hair cells but still present in adjacent support cells.
(7.40 MB EPS)

Text S1 Detailed methods for the construction of the Actb targeting construct and genotyping by Southern blot.
(0.04 MB DOC)

## References

[1] Herman IM (1993). Actin isoforms.. Curr Opin Cell Biol.

[2] Rubenstein PA (1990). The functional importance of multiple actin isoforms.. Bioessays.

[3] Bergeron SE, Zhu M, Thiem SM, Friderici KH, Rubenstein PA (2010). Ion-dependent polymerization differences between mammalian beta- and gamma-nonmuscle actin isoforms.. J Biol Chem.

[4] Bassell GJ, Zhang H, Byrd AL, Femino AM, Singer RH (1998). Sorting of beta-actin mRNA and protein to neurites and growth cones in culture.. J Neurosci.

[5] Dugina V, Zwaenepoel I, Gabbiani G, Clement S, Chaponnier C (2009). Beta- and gamma-cytoplasmic actins display distinct distribution and functional diversity.. Journal of Cell Science.

[6] Karakozova M, Kozak M, Wong CCL, Bailey AO, Yates JR (2006). Arginylation of beta-actin regulates actin cytoskeleton and cell motility.. Science.

[7] Shmerling D, Danzer C-P, Mao X, Boisclair J, Haffner M (2005). Strong and ubiquitous expression of transgenes targeted into the beta-actin locus by Cre/lox cassette replacement.. Genesis.

[8] Shawlot W, Deng JM, Fohn LE, Behringer RR (1998). Restricted beta-galactosidase expression of a hygromycin-lacZ gene targeted to the beta-actin locus and embryonic lethality of beta-actin mutant mice.. Transgenic Res.

[9] Belyantseva IA, Perrin BJ, Sonnemann KJ, Zhu M, Stepanyan R (2009). Gamma-actin is required for cytoskeletal maintenance but not development.. Proc Natl Acad Sci USA.

[10] Procaccio V, Salazar G, Ono S, Styers ML, Gearing M (2006). A mutation of beta-actin that alters depolymerization dynamics is associated with autosomal dominant developmental malformations, deafness, and dystonia.. Am J Hum Genet.

[11] Morell RJ, Friderici KH, Wei S, Elfenbein JL, Friedman TB (2000). A new locus for late-onset, progressive, hereditary hearing loss DFNA20 maps to 17q25.. Genomics.

[12] Rendtorff ND, Zhu M, Fagerheim T, Antal TL, Jones M (2006). A novel missense mutation in ACTG1 causes dominant deafness in a Norwegian DFNA20/26 family, but ACTG1 mutations are not frequent among families with hereditary hearing impairment.. Eur J Hum Genet.

[13] van Wijk E, Krieger E, Kemperman MH, De Leenheer EM, Huygen PL (2003). A mutation in the gamma actin 1 (ACTG1) gene causes autosomal dominant hearing loss (DFNA20/26).. J Med Genet.

[14] Zhu M, Yang T, Wei S, DeWan AT, Morell RJ (2003). Mutations in the gamma-actin gene (ACTG1) are associated with dominant progressive deafness (DFNA20/26).. Am J Hum Genet.

[15] Liu P, Li H, Ren X, Mao H, Zhu Q (2008). Novel ACTG1 mutation causing autosomal dominant non-syndromic hearing impairment in a Chinese family.. J Genet Genomics.

[16] Dallos P (1992). The active cochlea.. J Neurosci.

[17] Tilney LG, Derosier DJ, Mulroy MJ (1980). The organization of actin filaments in the stereocilia of cochlear hair cells.. J Cell Biol.

[18] Furness DN, Katori Y, Mahendrasingam S, Hackney CM (2005). Differential distribution of beta- and gamma-actin in guinea-pig cochlear sensory and supporting cells.. Hear Res.

[19] Slepecky NB, Savage JE (1994). Expression of actin isoforms in the guinea pig organ of Corti: muscle isoforms are not detected.. Hear Res.

[20] Frolenkov GI, Belyantseva IA, Friedman TB, Griffith AJ (2004). Genetic insights into the morphogenesis of inner ear hair cells.. Nat Rev Genet.

[21] Goodyear RJ, Marcotti W, Kros CJ, Richardson GP (2005). Development and properties of stereociliary link types in hair cells of the mouse cochlea.. J Comp Neurol.

[22] Ahmed ZM, Goodyear R, Riazuddin S, Lagziel A, Legan PK (2006). The tip-link antigen, a protein associated with the transduction complex of sensory hair cells, is protocadherin-15.. Journal of Neuroscience.

[23] Kazmierczak P, Sakaguchi H, Tokita J, Wilson-Kubalek EM, Milligan RA (2007). Cadherin 23 and protocadherin 15 interact to form tip-link filaments in sensory hair cells.. Nature.

[24] Keithley EM, Canto C, Zheng QY, Fischel-Ghodsian N, Johnson KR (2004). Age-related hearing loss and the ahl locus in mice.. Hear Res.

[25] Noben-Trauth K, Zheng QY, Johnson KR (2003). Association of cadherin 23 with polygenic inheritance and genetic modification of sensorineural hearing loss.. Nat Genet.

[26] Hébert JM, McConnell SK (2000). Targeting of cre to the Foxg1 (BF-1) locus mediates loxP recombination in the telencephalon and other developing head structures.. Dev Biol.

[27] Matei V, Pauley S, Kaing S, Rowitch D, Beisel KW (2005). Smaller inner ear sensory epithelia in Neurog 1 null mice are related to earlier hair cell cycle exit.. Dev Dyn.

[28] Höfer D, Ness W, Drenckhahn D (1997). Sorting of actin isoforms in chicken auditory hair cells.. J Cell Sci.

[29] Furness DN, Mahendrasingam S, Ohashi M, Fettiplace R, Hackney CM (2008). The dimensions and composition of stereociliary rootlets in mammalian cochlear hair cells: comparison between high- and low-frequency cells and evidence for a connection to the lateral membrane.. Journal of Neuroscience.

[30] Sonnemann KJ, Fitzsimons DP, Patel JR, Liu Y, Schneider MF (2006). Cytoplasmic gamma-actin is not required for skeletal muscle development but its absence leads to a progressive myopathy.. Developmental Cell.

[31] Wagner CR, Mahowald AP, Miller KG (2002). One of the two cytoplasmic actin isoforms in Drosophila is essential.. Proc Natl Acad Sci USA.

[32] Huttelmaier S, Zenklusen D, Lederer M, Dictenberg J, Lorenz M (2005). Spatial regulation of beta-actin translation by Src-dependent phosphorylation of ZBP1.. Nature.

[33] Kislauskis EH, Zhu X, Singer RH (1994). Sequences responsible for intracellular localization of beta-actin messenger RNA also affect cell phenotype.. J Cell Biol.

[34] Leung KM, van Horck FP, Lin AC, Allison R, Standart N (2006). Asymmetrical beta-actin mRNA translation in growth cones mediates attractive turning to netrin-1.. Nat Neurosci.

[35] Schneider ME, Belyantseva IA, Azevedo RB, Kachar B (2002). Rapid renewal of auditory hair bundles.. Nature.

[36] De La Cruz EM (2005). Cofilin binding to muscle and non-muscle actin filaments: isoform-dependent cooperative interactions.. J Mol Biol.

[37] Yao X, Cheng L, Forte JG (1996). Biochemical characterization of ezrin-actin interaction.. J Biol Chem.

[38] Namba Y, Ito M, Zu Y, Shigesada K, Maruyama K (1992). Human T cell L-plastin bundles actin filaments in a calcium-dependent manner.. J Biochem.

[39] Shuster CB, Lin AY, Nayak R, Herman IM (1996). Beta cap73: a novel beta actin-specific binding protein.. Cell Motil Cytoskeleton.

[40] Weber A, Nachmias VT, Pennise CR, Pring M, Safer D (1992). Interaction of thymosin beta 4 with muscle and platelet actin: implications for actin sequestration in resting platelets.. Biochemistry.

[41] Larsson H, Lindberg U (1988). The effect of divalent cations on the interaction between calf spleen profilin and different actins.. Biochim Biophys Acta.

[42] Tzima E, Trotter PJ, Orchard MA, Walker JH (2000). Annexin V relocates to the platelet cytoskeleton upon activation and binds to a specific isoform of actin.. Eur J Biochem.

[43] Johnson KR, Longo-Guess C, Gagnon LH, Yu H, Zheng QY (2008). A locus on distal chromosome 11 (ahl8) and its interaction with Cdh23 ahl underlie the early onset, age-related hearing loss of DBA/2J mice.. Genomics.

[44] Shin JB, Longo-Guess CM, Gagnon LH, Saylor KW, Dumont RA (2010). The R109H variant of fascin-2, a developmentally regulated actin crosslinker in hair-cell stereocilia, underlies early-onset hearing loss of DBA/2J mice.. J Neurosci.

[45] Bunnell TM, Ervasti JM (2010). Delayed embryonic development and impaired cell growth and survival in Actg1 null mice.. Cytoskeleton.

[46] Holzenberger M, Lenzner C, Leneuve P, Zaoui R, Hamard G (2000). Cre-mediated germline mosaicism: a method allowing rapid generation of several alleles of a target gene.. Nucleic Acids Res.

[47] Hanft LM, Rybakova IN, Patel JR, Rafael-Fortney JA, Ervasti JM (2006). Cytoplasmic gamma-actin contributes to a compensatory remodeling response in dystrophin-deficient muscle.. Proc Natl Acad Sci USA.

