# Supplementary material for: β-Actin and γ-Actin Are Each Dispensable for Auditory Hair Cell Development But Required for Stereocilia Maintenance
Source: PLoS Genet. 2010 Oct 14;6(10):e1001158. doi: 10.1371/journal.pgen.1001158 (PMC2954897; doi:10.1371/journal.pgen.1001158)
Supplement: Text S1 — Detailed methods for the construction of the Actb targeting construct and genotyping by Southern blot. (0.04 MB DOC) [file pgen.1001158.s004.doc]

**Text S1**

*Construction of targeting vector* - A mouse BAC clone encoding the entire β‑actin locus (*Actb*) was identified by PCR screening the RPCI-22 library (BACPAC Resource Center, Oakland, CA) and used to assemble the floxed *Actb* construct in the pKO NTKV 1901 plasmid (Stratagene, La Jolla, CA). A PCR-based approach was used to introduce loxP sites into the *Actb* allele by adding 34 bp loxP sequences to the 3’ end of oligonucleotide primers (Supplemental Figure 1A). Briefly, PCR amplification of BAC clone 4F10 with primers KJS82 (5’‑ctgagcagcttgtcactcccagaatcc‑3’) and KJS80 (5’‑*ggtacc*ataacttcgtatagcatacattatacgaagttataagcaagcttaaggcgctgg-3’) (loxP site is underlined) generated a 1.9 kb SpeI-KpnI fragment encoding exon 1 and novel loxP site “D” in intron I. This amplicon was co-inserted with a 4.5 kb *Actb* HindIII-SpeI fragment encoding upstream flanking sequence into the HindIII-KpnI sites of pKO NTKV 1901, producing pKO-ScAβ. PCR amplification of BAC clone 4F10 with primers KJS68 (5’‑*ggatcc*ataacttcgtataatgtatgctatacgaagttatggtaataatgcggccggtctg-3’) and KJS69 (5’‑*gaattc*aggataacttcgtatagcatacattatacgaagttatgagaaagggcgtggctgag-3’) generated a 0.7 kb *Actb* BamHI-EcoRI fragment encoding exons 2 and 3 floxed by sites “E” and “F”. This amplicon was co-inserted with a 2.1 kb *Actb* EcoRI-SalI fragment encoding exons 4, 5, and 6 into the BamHI‑SalI site of pKO-ScAβ, generating pKO‑ScABβ. The final pKO‑ScABβ construct consisted of a 4.5 kb 5’ homology arm, a neomycin cassette floxed by sites “D” and “E”, exons 2 and 3 floxed by “E” and “F”, and a 2.1 kb 3’ homology arm. All amplicons were first subcloned into pCR-Blunt (Invitrogen, Carlsbad, CA) and confirmed by sequencing before being excised. The entire pKO‑ScABβ construct was also confirmed by sequencing.

pKO‑ScABβ was linearized by NotI digestion and electroporated into 129S6 murine embryonic stem cells at the Gene Targeting Mouse Service Core (University of Cincinnati, Cincinnati, OH). Colonies surviving selection were expanded and screened by Southern blot analysis (see below for details). Clone T46 H127 was identified with the expected 5’ and 3’ recombination events and was subsequently karyotyped and injected into C57BL/6 blastocysts at the University of Wisconsin-Madison (Supplemental Figure 1B). Chimeric males were bred to C57BL/6 females to determine germline transmission of the floxed allele.

To remove the neomycin cassette from targeted mice, *Actb^flox+neo/+^* offspring were bred with mice that express Cre recombinase driven by the germline-expressed EIIa promoter [1] as described [2].

*Southern blot* - 5 μg of genomic DNA was digested overnight with excess HindIII and blotted as described [2]. A 190 bp probe complementary to sequence upstream of the targeting region was PCR amplified from BAC clone 4F10 using primers KJS88 (5’-agaagcttaccaccaagcctgatg-3’) and KJS89 (5’-ccactgaggcatctaacttctgacc-3’). A 240 bp probe complementary to sequence downstream of the targeting region was PCR amplified using primers KJS90 (5’-ccttcatggaaccccgtaaag-3’) and KJS91 (5’-tgtgggctgtaaacagttcgg-3’).

*Genotyping -* All genotype analyses of the *Actb* locus were performed by a multiplex PCR using a trio of primers on genomic tail DNA. Primers KJS110 (5’-ggtctggcttcctgccctaggtc-3’), KJS64 (5’-gtgaaactgtatggatagatctgagacatgc-3’), and KJS38 (5’-gctacttccatttgtcacgtcctgc-3’) were used in combination to discriminate between respective *Actb* alleles (Supplemental Figure 1C).

References

1. Holzenberger M, Lenzner C, Leneuve P, Zaoui R, Hamard G, et al. (2000) Cre-mediated germline mosaicism: a method allowing rapid generation of several alleles of a target gene. Nucleic Acids Res 28: E92.

2. Sonnemann KJ, Fitzsimons DP, Patel JR, Liu Y, Schneider MF, et al. (2006) Cytoplasmic gamma-actin is not required for skeletal muscle development but its absence leads to a progressive myopathy. Developmental Cell 11: 387-397.
